# Evaluation of the efficacy of a* Propionibacterium* extract gel in wound healing and symptom relief after open excisional hemorrhoidectomy: a randomized clinical study

**DOI:** 10.1007/s10151-026-03381-x

**Published:** 2026-06-23

**Authors:** S. Deidda, C. Eberspacher, V. Argiolas, L. Ventrone, A. Testa, S. Ascanelli, E. Rossin, G. Lisi, D. Mascagni, L. Zorcolo

**Affiliations:** 1 Colorectal Surgery Unit, Department of Surgical Science, A.O.U. Cagliari, University of Cagliari, Monserrato, S.S. 554 Bivio Sestu, 09042 Cagliari, Italy; 2 Department of Surgery, “Sapienza” University of Rome, Rome, Italy; 3 Chirurgia Generale, Ospedale San Pietro FBF, Rome, Italy; 4 Surgical Department, University Hospital, Ferrara, Italy; 5 Department of Surgery, Sant’Eugenio Hospital, Viale Dell’Umanesimo 10, Rome, Italy

**Keywords:** Hemorrhoidectomy, Postoperative pain, Topical therapy, Wound healing, Colorectal surgery, Patient satisfaction

## Abstract

**Background:**

Postoperative symptoms such as pain, burning, and itching are common following open excisional hemorrhoidectomy and are often associated with several factors, including wound healing. This study evaluated the efficacy of* Propionibacterium* extract gel (PeG) in promoting wound healing and reducing pain, burning, and itching compared to an ointment containing hyaluronic acid and silver sulfadiazine (HA-SSD).

**Methods:**

In this multicenter, phase IV randomized controlled trial, patients undergoing open excisional hemorrhoidectomy were randomly assigned in a 1:1 ratio to receive PeG or HA-SSD. The primary outcome (wound healing) was evaluated on the basis of grade of epithelialization. Secondary outcomes (pain, burning, and itching) were assessed using a 10-point visual analogue scale at baseline and 10, 20, and 40 days postoperatively.

**Results:**

Of the 119 screened patients, 64 (53.78%) received PeG and 55 (46.22%) received HA-SSD. The baseline characteristics were comparable. After 20 days, 46 (75.4%) of the PeG group patients had an epithelialization grade > 50% (vs *n* = 37, 72.6% of the HA-SSD group; *p* = 0.02). By day 40, complete wound healing occurred in 52 (85.3%) of the PeG group patients (vs *n* = 25, 52.1% of the HA-SSD group; *p* = 0.003). Both treatments reduced postoperative pain over time, with no significant between-group difference (*p* = 0.24). PeG demonstrated a superior reduction in burning (*p* = 0.02) and itching (*p* = 0.001). Patient satisfaction was higher with PeG (*n* = 45, 75% vs *n* = 9, 18.8% of the HA-SSD group, *p* < 0.001), with no reported adverse reactions.

**Conclusions:**

PeG was more efficient than HA-SSD in promoting wound healing, alleviating burning and itching, and enhancing patient satisfaction following hemorrhoidectomy. This study supports the safety and effectiveness of PeG as a therapeutic option for postoperative management.

**Clinical trial registration:**

NCT06872151, retrospectively registered on 6 March 2025.

**Supplementary Information:**

The online version contains supplementary material available at https://doi.org/10.1007/s10151-026-03381-x.

## Introduction

Hemorrhoidal disease (HD) is one of the most common proctological conditions, affecting approximately 50% of the population over 50 years old who have experienced symptoms that could impair their quality of life [1]. This condition affects patients between 45 and 65 years old with a prevalence rate of 4.4% [2]. Most patients with grade I or II HD, based on the Goligher classification [3], can be effectively managed with conservative therapy and outpatient procedures [2].

For patients who have not responded to conservative management and present with symptomatic grade III and IV HD [3], open excisional hemorrhoidectomy is considered the gold standard treatment [4]. Although this surgical procedure is effective, it carries a risk of postoperative complications that can significantly impact the patient’s quality of life. Complications following hemorrhoidectomy may include bleeding and anal stricture, which may necessitate re-intervention in both the short and long term [5]. Other common complications include postoperative pain, burning, and itching [6–8], resulting from multiple factors such as the degree of postoperative wound healing [9, 10].

Despite the availability of various therapeutic and surgical strategies such as radiofrequency and ultrasound devices [11, 12], and pedicle coagulation instead of ligation [10], to reduce discomfort after excisional hemorrhoidectomy and promote wound healing, these symptoms may persist in some patients. Few studies have analyzed the role of topical agents in alleviating these symptoms and aiding in the healing process [9, 13–15].

A previous study assessed the effectiveness of a gel containing a* Propionibacterium* extract gel (PeG; Emorsan® Gel; Depofarma S.p.A, Treviso, Italy) for pain control after open excisional hemorrhoidectomy [9]. The data demonstrated that pain levels were significantly lower in the PeG group, and the probability of wound healing was 66% higher in patients using PeG compared to those receiving conventional treatments.

Building on these findings, the present study aims to confirm these results and compare the effectiveness of PeG with an ointment containing hyaluronic acid and silver sulfadiazine (HA-SSD; Connettivina® Plus) which is well known for its efficacy in wound healing [16]. This study evaluates pain management and wound healing following open excisional hemorrhoidectomy, specifically assessing the clinical efficacy of PeG and HA-SSD in promoting wound epithelialization up to 40 days postsurgery.

## Methods

This study was an open-label randomized controlled trial conducted between November 2024 and April 2025, across five high-volume tertiary referral centers specializing in proctological diseases. The research received approval from the institutional review boards of each participating center (Protocol number 28209; October 16, 2024), in accordance with the principles of the Declaration of Helsinki (1996) and the International Conference on Harmonization Good Clinical Practice guidelines, and was registered on ClinicalTrials.gov in March 2025 (NCT06872151). Although trial registration occurred after enrollment had started, the study protocol, endpoints, and statistical analysis plan were prospectively defined before patient recruitment and were not modified during the study. Written informed consent was obtained from all participants involved in the study.

The study included all consecutive patients who underwent open excisional hemorrhoidectomy for Goligher III or IV degree [3] HD involving the removal of at least three hemorrhoidal piles, and who were aged between 18 and 75 years. Exclusion criteria encompassed patients who declined to provide consent, those with opioid dependence or chronic analgesic use, fecal incontinence, anorectal neoplasms, immunosuppressive treatments (e.g., chemotherapy, radiotherapy), chronic inflammatory bowel diseases, pregnancy, major psychiatric disorders, or known allergies to components of the treatments under investigation.

All surgical procedures were performed by specialized proctological surgeons. The Milligan–Morgan hemorrhoidectomy was conducted using the LigaSure system, without ligation of the vascular pedicle or concomitant internal sphincterotomy [10, 17, 18].

A block randomization design was used to allocate patients into two groups: the PeG group and HA-SSD group. PeG (30 g tube) is rich in dimethicone, cyclopentasiloxane, an antioxidant agent, and* Propionibacterium acnes* lysate; HA-SSD (25 g tube) contains hyaluronic acid and silver sulfadiazine.

Both treatments were administered in a similar manner, with patients instructed to apply 1 ml of gel (equivalent to one phalanx) twice daily for 20 consecutive days, manually to the anal and perianal areas, once in the morning after defecation and once in the evening before bedtime.

Patients were monitored at four time points: at baseline, defined as the day of the surgical procedure, corresponding to patients enrollment (visit 0), 10 days (visit 1), 20 days (visit 2), and 40 days (visit 3) following the surgical procedure. At each visit, data were collected on spontaneous pain and pain associated with defecation, burning, and itching using a 10-point visual analogue scale (VAS). The degree of wound epithelialization was assessed using three levels (1 to 3), corresponding to < 50%, > 50%, and 100% (complete healing), respectively. Data regarding bowel habits were evaluated using three patterns of the Bristol stool scale (hard, 1–2; normal, 3–5; non-formed, 6–7), along with the presence or absence of soiling and the sensation of incomplete evacuation. Patients were additionally asked to report the extent of activities they could undertake using a 3-item scale: limited (score 1), partially limited (score 2), and completely unlimited (score 3). At visit 3, patients were requested to indicate their level of satisfaction with the treatment using a 5-point rating scale (1, insufficient; 2, sufficient; 3, fair; 4, good; 5, excellent).

At each visit, it was necessary to document whether a digital rectal examination (DRE) was performed; anoscopy was conducted at both the enrollment visit and visit 3 (40 days postsurgery) to evaluate the degree of wound epithelialization. At enrollment, it was also critical to ascertain whether the patient had previously undergone a colonoscopy to exclude any neoplastic diseases.

Patients were permitted to discontinue their participation in the study prematurely for various reasons, including personal request without the need for justification. They could also withdraw if any adverse events occurred, if the investigator deemed it necessary to stop their participation, or if there were significant violations of the protocol, such as any conditions outlined in the exclusion criteria. Furthermore, patients were instructed to report any adverse events associated with the treatments and to provide descriptions of these events. The tolerability of the treatments was graded at visits 1–3 using a 6-point scale: 0, no erythema; 1, very mild erythema; 2, moderate erythema without edema; 3, moderate erythema with edema but without papules; 4, severe erythema with edema with or without papules; 5, severe erythema with edema and vesicles.

An analgesic regimen was prescribed, consisting of 1000 mg of paracetamol three times daily for 20 days. Additionally, patients were provided the option to take an additional analgesic (ketorolac drops), with dosage determined by the patient’s weight, for a maximum of two doses per day. During each visit, patients received a new diary to record the timing of analgesic intake and any additional doses taken each day for 10 days; the diary which was to be returned at the subsequent visit.

The primary objective of the study was to compare the efficacy of PeG and HA-SSD in promoting wound epithelialization during the postoperative period following open excisional hemorrhoidectomy. The primary endpoint was wound epithelialization, assessed at baseline and at 10, 20, and 40 days after treatment initiation using a three-level ordinal scale (≤ 50%, > 50%, and 100%). Secondary objectives included the evaluation of the efficacy of PeG and HA-SSD in alleviating postoperative pain, itching, and burning, all assessed by VAS.

### Sample size and randomization

The sample size was determined on the basis of a reference epithelialization rate exceeding 50% at 40 days post-treatment initiation in 38% of patients treated with the experimental treatment (PeG), in contrast to a proportion of patients treated with the reference treatment (HA-SSD) not exceeding 25% (odds ratio 2.45). This calculation was based on a design incorporating four repeated measures at intervals of 0, 10, 20, and 40 days [9].

With a desired power (1 − β) of 0.80 and assuming a compound symmetry covariance structure with an autocorrelation value between observations within the same patient not exceeding 0.50, an alpha level (α) of 0.10 necessitated a sample size of *N* = 55 patients per treatment arm; this culminated in a total sample size of *N* = 110 patients. To maintain the intended study power, the sample size was increased to compensate for a potential non-informative dropout rate not exceeding 10%. To ensure an equal distribution of patients across centers, 19 patients per center were targeted, resulting in a total enrollment of 114 patients.

At the outset of the study, patients were randomized, after surgery, to one of the two treatment groups, PeG and HA-SSD, utilizing a list generated through a block randomization design with blocks of size 6 (Table S1).

### Statistical analysis

All analyses were performed using SAS 9.4 (SAS Institute Inc., Cary, NC, USA). Data were presented using counts and percentages for categorical variables, and the mean with standard deviation (SD) for continuous variables. Patient characteristics were summarized by treatment arm. Results concerning primary and secondary endpoints were tabulated by treatment arm and visit (at baseline, visits 1, 2, and 3—at 10, 20, and 40 days from baseline, respectively). Individual profiles and mean plots over time and by treatment were generated for continuous variables, and bar graphs were used for categorical variables.

The normality assumption for continuous variables was evaluated using the Shapiro–Wilk test. Between-treatment differences for categorical variables were assessed using Fisher’s exact test, whereas continuous variables were analyzed using either the unpaired* t* test or the Wilcoxon two-sample test, as appropriate. For continuous variables, a repeated measures mixed model was also fitted, incorporating time, treatment, and the treatment-by-time interaction as fixed effects, with patient ID included as a random effect.

The primary endpoint has been analyzed through a Fisher’s exact test considering three epithelialization categories (≤ 50%, > 50%, 100%) for each time point. A post hoc power analysis was additionally performed to estimate the achieved statistical power for the primary endpoint at 40 days follow-up, based on the observed effect size and the available sample size.

Considering the paucity of missing data, they were handled through listwise deletion. Although the sample size calculation assumed *α* = 0.10, all inferential analyses were performed using a two-tailed *α* = 0.05 to adopt a more conservative control of type I error. Results with 0.05 ≤ *p* < 0.10 are described as trend-level findings.

## Results

### Patient characteristics

**Fig. 1:**
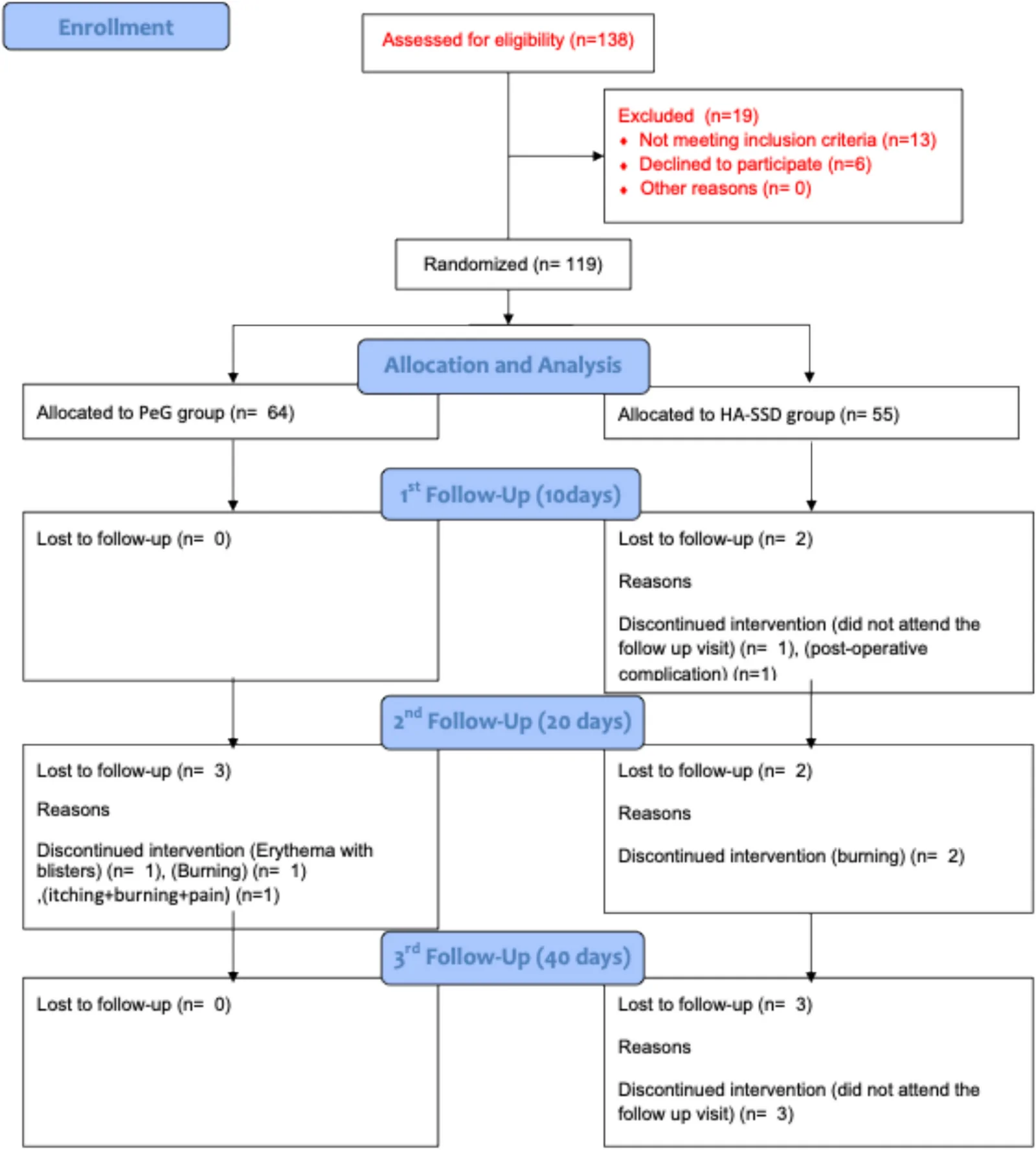
CONSORT diagram

Figure 1 and Table 1 present the CONSORT diagrams [16] and patient characteristics at baseline and treatment days, respectively. No statistically significant differences were observed between the treatment arms for any of the baseline variables. A total of 64 (53.78%) patients were assigned to the PeG group, whereas 55 (46.22%) patients were allocated to the HA-SSD group. A total of three patients discontinued treatment with PeG and were not assessed at 20 days follow-up, while in the HA-SSD arm two patients were lost at first and second follow-up and three additional patients at last follow-up. The reasons leading to dropout are specified in Fig. 1. Overall, the number of patients assessed at 10, 20, and 40 days post-baseline was 53, 51, and 48 for HA-SSD, and 64, 61, and 61 for PeG, respectively.

**Table 1:** Patients’ characteristics at baseline by treatment arm (*n* = 119)

Characteristic^a^	Total *n* = 119	HA-SSD *n* = 55 (46.22%)	PeG *n* = 64 (53.78%)	*p* value^b^
Age, years	50.6 (12.7)	51.3 (13.5)	50.1 (12.0)	0.63
Treatment days	19.2 (6.3)	19.2 (8.2)	19.2 (4.1)	0.51
Sex				1.00
Female	51 (42.9)	24 (43.6)	27 (42.2)	
Male	68 (57.1)	31 (56.4)	37 (57.8)	
Pregnancy				0.25
Nulliparous	17 (14.3)	10 (41.7)	7 (25.9)	
Multipara	34 (28.6)	14 (58.3)	20 (74.1)	

^a^Numbers indicate mean (standard deviation) for age and treatment days, and *N* (%) based on non-missing values for the other variables

^b^Unpaired *t* test for age, Wilcoxon test for treatment days, Fisher’s exact test otherwise

### Epithelialization

**Fig. 2:**
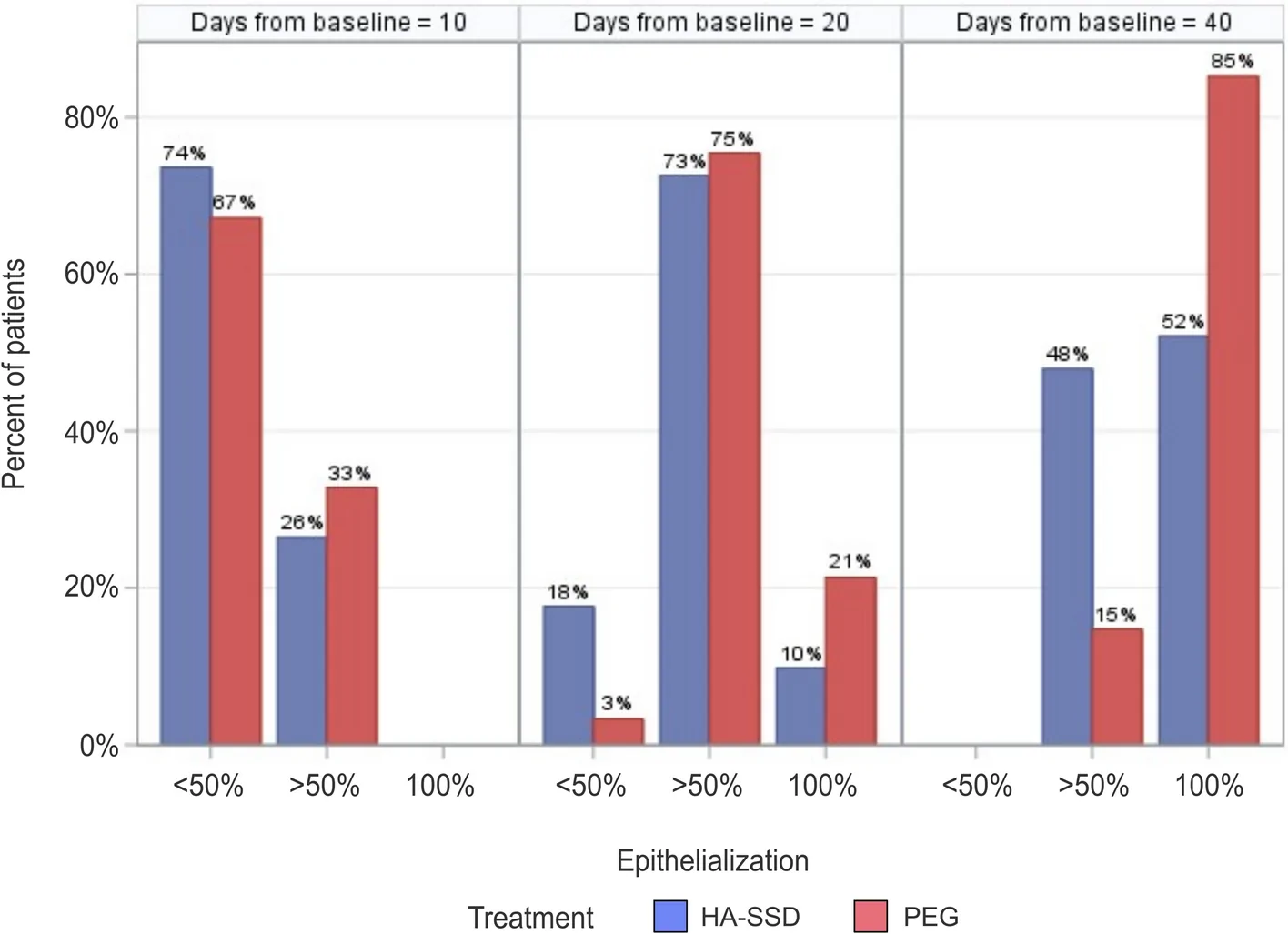
Epithelialization grade by time and treatment arm

Significant differences in the distribution of epithelialization grades between HA-SSD and PeG were observed at 20 and 40 days. After 10 days, 21 (32.8%) patients in the PeG group achieved an epithelialization greater than 50% compared to 14 (26.4%) in the HA-SSD group (*p* = 0.54). Complete epithelialization was significantly more frequent in the PeG group after 20 days (*n* = 13, 21.3% vs *n* = 5, 9.8%; *p* = 0.02), and after 40 days (*n* = 52, 85.3% vs *n* = 25, 52.1%; *p* = 0.003) (Table 2, Fig. 2).

**Table 2:** Frequency distribution of epithelialization levels stratified by time point and treatment arm

Days from baseline	Epithelialization	All patients	Treatment, *n* (%)		*p* value^a^
			HA-SSD	PeG	
10	≤ 50%	82 (70.1)	39 (73.6)	43 (67.2)	0.54
	> 50%	35 (29.9)	14 (26.4)	21 (32.8)	
	100%	0	0	0	
	Total	117	53	64	
20	≤ 50%	11 (9.8)	9 (17.7)	2 (3.3)	0.02
	> 50%	83 (74.1)	37 (72.6)	46 (75.4)	
	100%	18 (16.1)	5 (9.8)	13 (21.3)	
	Total	112	51	61	
40	> 50%	32 (29.4)	23 (47.9)	9 (14.8)	0.003
	100%	77 (70.6)	25 (52.1)	52 (85.3)	
	Total	109	48	61	

^a^*p* values refer to Fisher’s exact tests

In a post hoc analysis, the statistical power associated with the observed difference at the 40-day follow-up was 90% at *α* = 0.05 and 95% at *α* = 0.10.

### VAS for basal pain

VAS for basal pain showed a similar trend over time in both treatment groups with a maximum after 10 days, followed by a progressive decrease at 20 and 40 days. No statistically significant differences between the two treatment groups were observed at any time point. However, a lower mean of VAS score was observed in the PeG arm after 20 [2.6 (± 2.5) vs 3.4 (± 2.5); *p* = 0.09) and 40 days [0.9 (± 1.7) vs 1.3 (± 2.5); *p* = 0.10). Overall, treatment had no significant effect on basal pain VAS scores (*p* = 0.24) (Tables 3 and S2; Fig. S1b).

**Table 3:** Summary statistics for VAS scores reported for basal pain, burning, itching, and defecation pain by time and treatment arm

Score^a^	Days from baseline	All patients^a^	Treatment^a^		*p* value^b^
			HA-SSD	PeG	
VAS basal pain	0	2.7 (2.9)	2.9 (2.9)	2.6 (2.9)	0.47
	10	5.0 (2.7)	5.5 (2.4)	4.7 (2.8)	0.17
	20	3.0 (2.5)	3.4 (2.5)	2.6 (2.5)	0.09
	40	1.1 (1.7)	1.3 (2.5)	0.9 (1.7)	0.10
	Overall	2.9 (2.9)	3.3 (1.6)	2.7 (2.9)	0.24
VAS burning	0	3.8 (2.9)	3.7 (2.7)	3.8 (3.0)	0.85
	10	4.4 (2.9)	4.7 (2.9)	4.1 (2.9)	0.22
	20	2.2 (2.1)	2.8 (2.1)	1.7 (2.0)	0.005
	40	0.7 (1.1)	1.2 (1.2)	0.4 (1.0)	< 0.001
	Overall	2.8 (2.8)	3.1 (2.7)	2.6 (2.8)	0.02
VAS itching	0	3.1 (3.2)	2.8 (3.1)	3.4 (3.3)	0.36
	10	3.1 (2.6)	3.6 (2.8)	2.6 (2.4)	0.08
	20	1.9 (2.0)	2.3 (2.1)	1.6 (1.9)	0.03
	40	0.7 (1.4)	1.0 (1.3)	0.5 (1.4)	0.007
	Overall	2.6 (2.6)	2.5 (2.6)	2.0 (2.6)	0.001
VAS defecation pain	0	3.1 (3.1)	3.4 (3.3)	2.9 (3.2)	0.45
	10	6.4 (2.5)	6.6 (2.5)	6.3 (2.5)	0.25
	20	4.3 (2.4)	4.9 (2.1)	3.7 (2.5)	0.004
	40	1.6 (1.7)	1.9 (1.5)	1.3 (1.8)	0.02
	Overall	3.9 (3.1)	4.3 (3.0)	3.6 (3.1)	0.09

^a^Data are reported as mean (standard deviation)

^b^Between treatment within visit comparison

### VAS for burning

VAS for burning showed similar variations over time in both treatment groups; the highest mean for burning was observed at 10 days, followed by a progressive decline after 20 and 40 days. While no significant differences were observed at baseline and 10 days (*p* = 0.85 and *p* = 0.22, respectively), scores were significantly lower in the PeG group at 20 days [1.7 (± 2.0) vs 2.8 (± 2.1); *p* = 0.005] and 40 days [0.4 (± 1.0) vs 1.2 (± 1.2); *p* < 0.001]. Overall, PeG treatment resulted in significantly lower burning pain scores compared with HA-SSD [2.6 (± 2.8) vs 3.1 (± 3.7); *p* = 0.02] (Tables 3 and S2; Fig. S2a, b).

### VAS for itching

VAS scores for itching showed no significant differences at baseline (*p* = 0.36) and a slight difference at 10 days (*p* = 0.08). At 20 days, scores were significantly lower in the PeG group compared with HA-SSD group [1.6 (± 1.9) vs 2.3 (± 2.1); *p* = 0.03], with a significant difference persisting at 40 days [0.5 (± 1.4) vs 1.0 (± 1.3); *p* = 0.007]. Overall, PeG treatment resulted in significantly lower itching scores than HA-SSD [2.0 (± 2.6) vs 2.5 (± 2.6); *p* = 0.001] (Tables 3 and S2; Fig. S3a, b).

### Defecation pain

VAS scores for defecation pain followed a similar trend over time in both treatment groups, with a peak at 10 days. No significant differences were observed at baseline (*p* = 0.45) or at 10 days (*p* = 0.25). At 20 days, scores were significantly lower in the PeG group compared with HA-SSD group [3.7 (± 2.5) vs 4.9 (± 2.1); *p* = 0.004], with the difference remaining significant at 40 days [1.3 (± 1.8) vs 1.9 (± 1.5); *p* = 0.02]. Overall, the difference between treatments reached slight statistical significance [3.6 (± 3.1) vs 4.3 (± 3.0); *p* = 0.09) (Tables 3 and S2; Fig. S4a).

### Bowel function

Bowel function was similar in both groups at baseline. After 10 days a significantly higher proportion of patients in the PeG group had normal bowel function (*n* = 49, 77.8% vs *n* = 31, 58.5%; *p* = 0.04). After 20 days, most patients showed normal bowel function, with a trend toward higher constipation in HA-SSD group (*n* = 2, 3.3 vs *n* = 8, 15.7; *p* = 0.07). By day 40, a significantly higher rate of normal bowel function was observed in the PeG group (*n* = 57, 95% vs *n* = 38, 79.2%; *p* = 0.02), with persistent constipation significantly more frequent in HA-SSD group. Diarrheal events remained rare (< 10%) throughout (Table S3).

### Health status

**Fig. 3:**
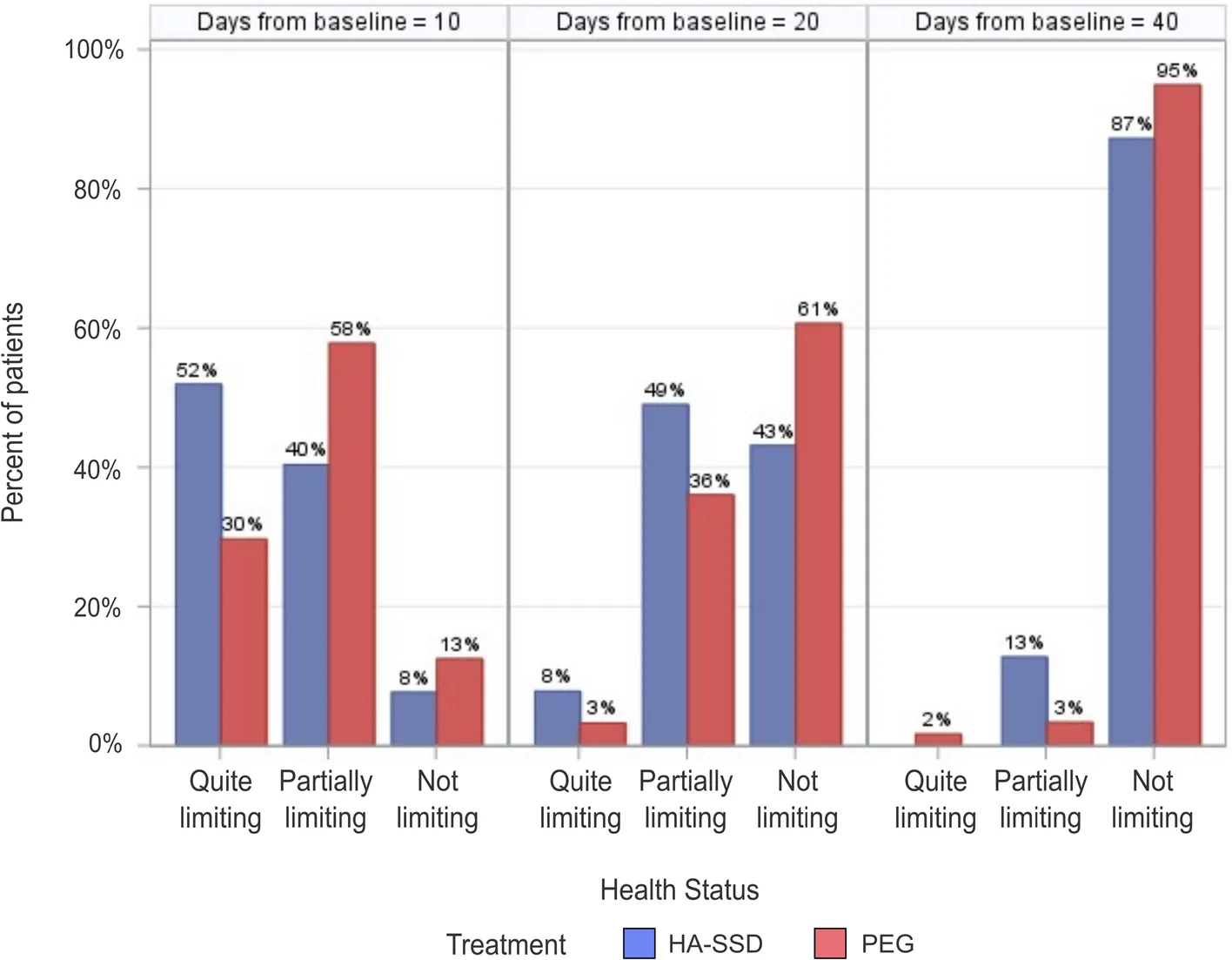
Health status by time and treatment arm

Health status demonstrated an overall increase from a “quite limiting” to “no limiting” condition, rising from 10.3% to 91.5% at 10 and 40 days, respectively, with some higher, albeit not significant, proportions favoring PeG at any time point (Table 4, Fig. 3).

**Table 4:** Health status by time and treatment arm

Days from baseline	Health status	All patients	Treatment, *n* (%)^a^		*p* value^c^
			HA-SSD	PeG	
10	Quite limiting	46 (39.7)	27 (51.9)	19 (29.7)	0.05
	Partially limiting	58 (50.0)	21 (40.4)	37 (57.8)	
	Not limiting	12 (10.3)	4 (7.7)	8 (12.5)	
	Missing^b^	1 (0.9)	1 (1.9)	0	0.45
	Total	117	53	64	
20	Quite limiting	6 (5.4)	4 (7.8)	2 (3.3)	0.15
	Partially limiting	47 (42.0)	25 (49.0)	22 (36.1)	
	Not limiting	59 (52.7)	22 (43.1)	37 (60.7)	
	Total	112	51	61	
40	Quite limiting	1 (0.9)	0	1 (1.7)	0.13
	Partially limiting	8 (7.6)	6 (12.8)	2 (3.4)	
	Not limiting	97 (91.5)	41 (87.2)	56 (94.9)	
	Missing^b^	3 (2.8)	1 (2.1)	2 (3.3)	1.00
	Total	118	48	61	

^a^Percent based on non-missing values

^b^Percent based on total number of patients

^c^Fisher’s exact test

### Tolerability

**Fig. 4:**
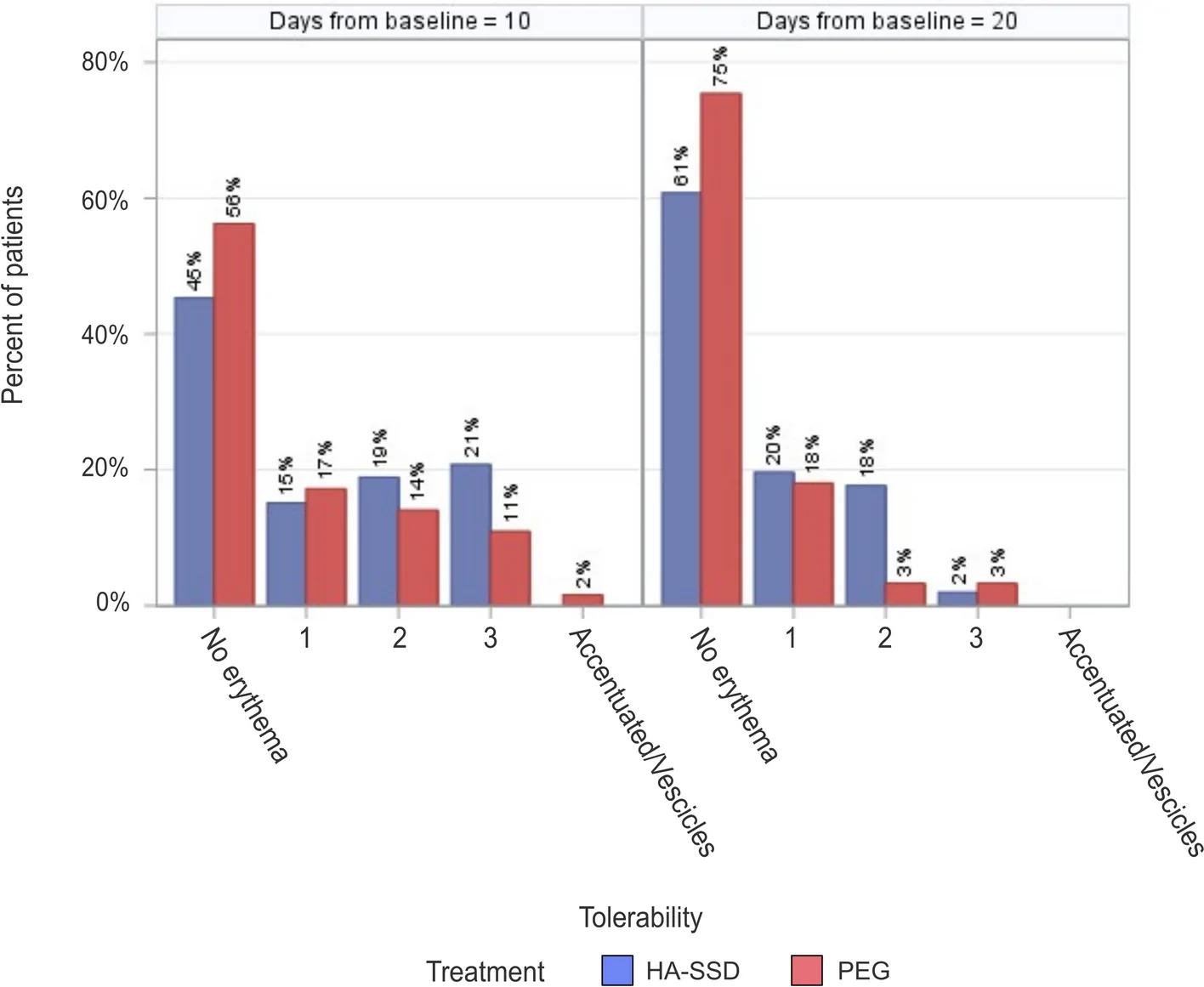
Tolerability score distribution by time and treatment arm

Tolerability scores were generally favorable in both groups. At day 10, over half of the patients reported no erythema (score 0), with similar distributions between the groups (*p* = 0.44). By day 20, the proportion of patients without erythema increased in both groups, reaching 75.4% in the PeG group and 60.8% in the HA-SSD group, showing a trend toward better tolerability with PeG (*p* = 0.06) (Table 5, Fig. 4).

**Table 5:** Tolerability score frequency distribution by time and treatment arm

Days from baseline	Tolerability score^§^	All patients	Treatment, *N* (column %)^a^		*p* value^b^
			HA-SSD	PeG	
10	0	60 (51.3)	24 (45.3)	36 (56.3)	0.44
	1	19 (16.2)	8 (15.1)	11 (17.2)	
	2	19 (16.2)	10 (18.8)	9 (14.1)	
	3	18 (15.4)	11 (20.8)	7 (10.9)	
	5	1 (0.9)	0	1 (1.6)	
	Total	117	53	64	
20	0	77 (68.8)	31 (60.8)	46 (75.4)	0.06
	1	21 (18.8)	10 (19.6)	11 (18.0)	
	2	11 (9.8)	9 (17.7)	2 (3.3)	
	3	3 (2.7)	1 (2.0)	2 (3.3)	
	Total	112	51	61	

^a^Percent based on non-missing values

^b^Percent based on total number of patients

^c^Fisher’s exact test

^§^0 = No erythema, 5 = Accentuated erythema with vesicles

### Adverse events

A total of 20 adverse events (AEs) was observed (15 in the HA-SSD group, 5 in the PeG group): 11 (9.4%) events occurred at 10 days, 6 (5.4%) at 20 days and 3 (2.8%) at 40 days follow-up. A significantly lower proportion of adverse events was observed in the PeG group compared to the HA-SSD group at 20 days (*n* = 0, 0% vs *n* = 6, 11.8%, *p* = 0.008) (Table 6). Specifically, two patients (3.9%) in the HA-SSD group reported rectal bleeding, and three patients (5.9%) reported itching. The distribution of adverse events by type is detailed in Table S4.

**Table 6:** Adverse events type frequency distribution by treatment arm and time

Days from baseline	*N*	All patients	Treatment, *N* (%)^*a*^		*p* value^b^
			HA-SSD	PeG	
10	117	11 (9.4)	7 (13.2)	4 (6.3)	0.22
20	112	6 (5.4)	6 (11.8)	0	0.008
40	108	3 (2.8)	2 (4.3)	1 (1.6)	0.58

^a^Percent based on non-missing data

^b^Fisher’s exact test

### Paracetamol and ketorolac consumption

The average number of paracetamol pills per patient significantly decreased over time for both treatment groups (*p* < 0.001), with no significant overall difference between treatments (*p* = 0.73) at any time point (Tables 7 and S5; Fig. S5). At baseline, the mean number of pills per day was 2.5 (± 0.7) for the HA-SSD group and 2.2 (± 0.8) for the PeG group (*p* = 0.08). By 40 days, this had decreased to 0.8 (± 0.6) vs 0.7 (± 0.6), respectively (*p* = 0.28). The average number of ketorolac drops consumed per patient was significantly lower for the PeG group at 10 days [8.2 (± 10.3) vs 11.8 (± 10.6), *p* = 0.02]. However, by 40 days, the difference was no longer significant [5.2 (± 7.4) vs 4.3 (± 8.7), *p* = 0.23] (Fig. S6).

**Table 7:** Average number of pills/patient summary statistics by time and treatment arm

	Days from baseline	*N*	Treatment^a^			*p* value^b^
			HA-SSD	*N*	PeG	
Paracetamol	10	53	2.5 (0.7)	64	2.2 (0.8)	0.08
	20	51	1.6 (0.9)	61	1.5 (1.0)	0.18
	40	48	0.8 (0.6)	61	0.7 (0.6)	0.28
	Overall		1.7 (1.6)		1.5 (1.0)	0.73
Ketorolac	10	53	11.8 (10.6)	64	8.2 (10.3)	0.02
	20	51	5.2 (7.4)	61	4.3 (8.7)	0.23
	40	–	–	–	–	–
	Overall		8.6 (9.7)		6.3 (9.7)	0.28

^a^Mean (standard deviation)

^b^*p* values refer to Wilcoxon test

### Treatment satisfaction

Treatment satisfaction was significantly different between the two groups (*p* < 0.001). A higher proportion of patients allocated to PeG group reported being extremely satisfied compared to the those in the HA-SSD group (*n* = 45, 75% vs *n* = 9, 18.8%; *p* < 0.001). Conversely, 7 patients (14.6%) in the HA-SSD group expressed neutrality or dissatisfaction regarding the treatment (Table 8).

**Table 8:** Treatment satisfaction at 40 days from baseline by treatment arm

Satisfaction grade	All patients *N* = 108	Treatment, *n* (%)^a^		*p* value^c^
		HA-SSD *N* = 48	PeG *N* = 60	
Unsatisfied	1 (0.9)	1 (2.1)	0	< 0.001
Neutral	6 (5.6)	6 (12.5)	0	
Quite satisfied	20 (18.5)	15 (31.3)	5 (8.3)	
Very satisfied	27 (25.0)	17 (35.4)	10 (16.7)	
Extremely satisfied	54 (50.0)	9 (18.8)	45 (75.0)	
Missing^b^	11 (9.2)	7 (12.7)	4 (6.3)	0.34

^a^Percent based on non-missing values

^b^Percent based on total number of patients

^c^Fisher’s exact test

## Discussion

The results of our study suggest PeG may offer benefits in the postoperative management of patients undergoing open excisional hemorrhoidectomy. To our knowledge, this represents the first randomized clinical trial that evaluates the efficacy of PeG compared to HA-SSD in enhancing postoperative recovery following open excisional hemorrhoidectomy.

The study aimed to investigate the efficacy of PeG versus HA-SSD in promoting epithelialization after open excisional hemorrhoidectomy. Assessments at baseline, 10, 20, and 40 days showed that PeG led to significantly better epithelialization at 20 and 40 days. By the end of the study, 85.3% of patients treated with PeG achieved complete epithelization, compared to 52.1% in the HA-SSD group.

To our knowledge, only one prospective study has previously assessed the efficacy of PeG compared with standard care following open excisional hemorrhoidectomy, particularly regarding pain management [9]. The authors reported complete wound healing at the final follow-up in all patients treated with PeG, compared to only 68% in the control group. In the multivariate analysis, the likelihood of wound healing was 66% higher in the PeG group, although this did not reach statistical significance. While the designs and comparators differ, both studies point toward a potential beneficial effect of PeG on postoperative wound healing.

In contrast to that study, ours is distinguished by the inclusion of a control group treated with HA-SSD, a commonly utilized product in clinical practice, recognized for its beneficial effects on tissue regeneration and re-epithelialization [19, 20]. The presence of hyaluronic acid in HA-SSD supports wound healing, while silver sulfadiazine provides antimicrobial activity. The high bacterial load in the anal canal warrants the use of topical antibiotics post-hemorrhoidectomy to prevent wound infection. However, some studies [21–23] have indicated that wound infection following hemorrhoidectomy is uncommon (approximately 1.7%), and this high bacterial load does not impair wound healing despite the presence of facultative pathogens. Furthermore, reducing the bacterial load at the hemorrhoidectomy wound through topical antibiotics does not enhance wound healing or decrease local infection rates [24]. In this context, the present results suggest that a non-antibiotic topical agent such as PeG may represent an effective alternative for postoperative wound care.

A recent study [14] described a combination of physiological mechanisms associated with the ability of PeG to accelerate wound healing. The antioxidative properties of a gel containing a* Propionibacterium* extract, which inhibits radical oxygen species and nitrogen species, were highlighted in an in vitro test. This study subsequently conducted an in vivo experiment using a mouse model and demonstrated that PeG increased the speed and percentage of wound area closure by inhibiting free radicals, thus potentially reducing local inflammation and oxidative stress.

Other studies have investigated alternatives agents after open excisional hemorrhoidectomy. Topical sucralfate has been shown to alleviate pain, improve wound healing, and minimize the usage of anti-inflammatory drugs compared to placebo [25, 26]. Furthermore, glyceryl trinitrate ointment has been reported to decrease postoperative pain and improve wound healing at 3 weeks compared with placebo [27]. Topical metronidazole may be effective and safe in decreasing postoperative pain in patients undergoing hemorrhoidectomy, although the effects on epithelialization are less clear [28]. While these studies suggest potential benefits of alternative topical treatments, the available evidence is limited, and direct comparisons with PeG are lacking. However, in our study PeG was generally well tolerated, with only a few adverse events reported.

Our results indicated that wound healing is closely associated with other common postoperative symptoms experienced by patients following open hemorrhoidectomy, such as pain, itching, burning sensation, and bowel habit variations, which may be associated with inflammation affecting mucocutaneous bridges and representing a frequent cause of discomfort. Although the mean VAS score for baseline pain did not demonstrate statistically significant improvement over time in the PeG group, patients reported reduced discomfort in terms of burning and itching during the initial 10 days, accompanied by a notable decrease in the consumption of paracetamol and ketorolac. Moreover, the VAS score for defecation-related pain was lower at 20 and 40 days in the PeG group, suggesting a potential reduction in inflammation and decreased need for analgesics, which may further corroborate the beneficial effects on wound healing.

The observed improvement in epithelialization with PeG compared to HA-SSD may offer potential advantages, such as faster recovery and reduced postoperative discomfort. These findings may guide future clinical research, and, if confirmed in larger studies, PeG could be considered as an option for promoting wound healing following open excisional hemorrhoidectomy. Furthermore, if PeG decreases healing duration and reduces complication rates, it could result in substantial cost savings within the healthcare system, including fewer follow-up visits, dressing changes, and secondary treatments. This not only stands to benefit patients but may also promote more efficient resource utilization in clinical environments. If confirmed by larger trials, our findings could have profound implications for the postoperative management of hemorrhoidal disease.

However, this study has several limitations. First, it was not a blinded study; neither the patients nor the investigators were blinded to treatment allocation, which may have introduced performance or assessment bias. Wound was assessed clinically without standardized or objective criteria, potentially introducing subjectivity and variability in the assessment. Furthermore, no formal interobserver agreement was established for the assessment of re-epithelialization, which may have impacted the consistency of outcome assessment. The absence of a no-treatment arm represents a further methodological limitation, as the contribution of spontaneous healing to the observed outcomes cannot be entirely excluded. A three-arm design would represent a methodologically stronger approach and is recommended for future investigations. Moreover, secondary endpoints should be regarded as purely explorative in nature as the sample size was not defined on the basis of these outcomes and no correction for multiple comparisons or hierarchical testing strategy has been applied.

However, the principal strength of this study lies in its randomized controlled trial design and the inclusion of a control group treated with a commonly employed topical agent, thereby facilitating a meaningful comparison with a product already integrated in clinical practice. Symptom assessment and patient satisfaction were evaluated using VAS scales, which are practical, but also less precise than validated disease-specific patient-reported outcome measures and quality of life questionnaires, the adoption of which is recommended in future trials.

## Conclusion

Emorsan® gel showed a trend toward improved outcome compared with Connettivina^®^ Plus in terms of wound healing, reducing postoperative burning and itching, and improving patient satisfaction following open hemorrhoidectomy. These results suggest that PeG is generally safe and effective in the postoperative recovery process after open excisional hemorrhoidectomy. If validated through further research, these findings suggest that PeG may support accelerated healing, decrease analgesic requirements, and help reduce the use of antibiotic topical agents.

## Supplementary Information

Below is the link to the electronic supplementary material.

Supplementary file1 (DOCX 29 kb)

## Data Availability

The data that support the findings of this study are available from the corresponding author upon reasonable request.
