# Supplementary material for: Evaluation of the efficacy of a Propionibacterium extract gel in wound healing and symptom relief after open excisional hemorrhoidectomy: a randomized clinical study
Source: Tech Coloproctol. 2026 Jun 23;30(1):142. doi: 10.1007/s10151-026-03381-x (PMC13547177; doi:10.1007/s10151-026-03381-x)
Supplement: Supplementary file 1 [file 10151_2026_3381_MOESM1_ESM.docx]

**Evaluation of the efficacy of a Propionibacterium extract gel in wound healing and symptom relief after open excisional hemorrhoidectomy: A randomized clinical study**

Simona Deidda, MD^1*^; Chiara Eberspacher, MD, PhD^2^; Valerio Argiolas, MD^1^; Luca Ventrone, MD^2^; Alessandro Testa, MD^3^; Simona Ascanelli, MD^4^; Eleonora Rossin, MD^4^; Giorgio Lisi, MD^5^; Domenico Mascagni, MD ^2^; Luigi Zorcolo, MD^1^

^1^ Colorectal Surgery Unit, A.O.U. Cagliari, Department of Surgical Science, University of Cagliari, Italy

^2^ Department of Surgery, “Sapienza” University of Rome, Italy

^3^ Chirurgia Generale, Ospedale San Pietro FBF, Rome, Italy

^4^ Surgical Department University Hospital Ferrara Italy

^5^ Department of Surgery, Sant'Eugenio Hospital, Viale Dell'Umanesimo 10, Rome, Italy

***Correspondence to:**

Dr. Simona Deidda, MD

Colorectal Surgery Unit, A.O.U. Cagliari,

Department of Surgical Science,

University of Cagliari, Monserrato,

S.S. 554 Bivio Sestu, Cagliari 09042, Italy

**e-mail:** [simona.deidda86@unica.it](mailto:simona.deidda86@unica.it)

**orcid:** <https://orcid.org/0000-0002-7756-9727>

**Techniques in Coloproctology**

| **Table S1.** Patient’s characteristic and procedures at baseline by treatment arm for N = 119 patients | |
| --- | --- |
| **N° paziente** | **Trattamento** |
| 001 | HA-SSD |
| 002 | HA-SSD |
| 003 | PEG |
| 004 | PEG |
| 005 | PEG |
| 006 | HA-SSD |
| 007 | PEG |
| 008 | HA-SSD |
| 009 | PEG |
| 010 | HA-SSD |
| 011 | PEG |
| 012 | HA-SSD |
| 013 | PEG |
| 014 | PEG |
| 015 | HA-SSD |
| 016 | PEG |
| 017 | HA-SSD |
| 018 | HA-SSD |
| 019 | HA-SSD |
| 020 | PEG |
| 021 | PEG |
| 022 | PEG |
| 023 | HA-SSD |
| 024 | HA-SSD |
| 025 | PEG |
| 026 | HA-SSD |
| 027 | PEG |
| 028 | HA-SSD |
| 029 | HA-SSD |
| 030 | PEG |
| 031 | HA-SSD |
| 032 | HA-SSD |
| 033 | PEG |
| 034 | PEG |
| 035 | PEG |
| 036 | HA-SSD |
| 037 | HA-SSD |
| 038 | HA-SSD |
| 039 | PEG |
| 040 | HA-SSD |
| 041 | PEG |
| 042 | PEG |
| 043 | HA-SSD |
| 044 | PEG |
| 045 | HA-SSD |
| 046 | HA-SSD |
| 047 | PEG |
| 048 | PEG |
| 049 | PEG |
| 050 | PEG |
| 051 | HA-SSD |
| 052 | HA-SSD |
| 053 | PEG |
| 054 | HA-SSD |
| 055 | HA-SSD |
| 056 | PEG |
| 057 | PEG |
| 058 | HA-SSD |
| 059 | HA-SSD |
| 060 | PEG |
| 061 | PEG |
| 062 | HA-SSD |
| 063 | PEG |
| 064 | PEG |
| 065 | HA-SSD |
| 066 | HA-SSD |
| 067 | PEG |
| 068 | PEG |
| 069 | HA-SSD |
| 070 | HA-SSD |
| 071 | HA-SSD |
| 072 | PEG |
| 073 | HA-SSD |
| 074 | PEG |
| 075 | PEG |
| 076 | HA-SSD |
| 077 | PEG |
| 078 | HA-SSD |
| 079 | HA-SSD |
| 080 | HA-SSD |
| 081 | PEG |
| 082 | PEG |
| 083 | PEG |
| 084 | HA-SSD |
| 085 | PEG |
| 086 | HA-SSD |
| 087 | HA-SSD |
| 088 | HA-SSD |
| 089 | PEG |
| 090 | PEG |
| 091 | HA-SSD |
| 092 | PEG |
| 093 | PEG |
| 094 | HA-SSD |
| 095 | HA-SSD |
| 096 | PEG |
| 097 | HA-SSD |
| 098 | PEG |
| 099 | HA-SSD |
| 100 | PEG |
| 101 | HA-SSD |
| 102 | PEG |
| 103 | HA-SSD |
| 104 | PEG |
| 105 | PEG |
| 106 | PEG |
| 107 | HA-SSD |
| 108 | HA-SSD |
| 109 | HA-SSD |
| 110 | PEG |
| 111 | PEG |
| 112 | HA-SSD |
| 113 | HA-SSD |
| 114 | PEG |

**Table S2**. Summary of the repeated measures mixed models conducted on VAS scores (basal pain, burning, itching and defecation pain) and the average number of pills/patient (Paracetamol and Ketorolac). The models incorporated time, treatment, and the treatment-by-time interaction as fixed effects, with patient ID included as a random effect.

| **Mixed models** | *Baseline score* | *Treatment x Time* | *Treatment* | *Days from baseline* |
| --- | --- | --- | --- | --- |
| *VAS Scores* | | | | |
| Basal pain | p < 0.001 | p = 0.57 | p = 0.24 | p < 0.001 |
| Burning | p < 0.001 | p = 0.22 | p = 0.02 | p < 0.001 |
| Itching | p < 0.001 | p = 0.02 | p = 0.001 | p < 0.001 |
| Defecation pain | p < 0.001 | p = 0.42 | p = 0.09 | p < 0.001 |

**Table S3.** Bowel Function by time by treatment arm

|  |  |  | **Treatment, N (column %) *^a^*** | |  |
| --- | --- | --- | --- | --- | --- |
| **Days from baseline** | **Bowel function** | **All patients**  **n=119** | **HA-SSD**  **n= 55** | **PeG**  **n= 64** | **p-value*^c^*** |
| **0** | **Diarrhoeal** | 9 (7.6) | 5 (9.1) | 4 (6.3) |  |
|  | **Normal** | 60 (50.4) | 24 (43.6) | 36 (56.2) |  |
|  | **Constipated** | 50 (42.0) | 26 (47.3) | 24 (37.5) | 0.40 |
|  | **Total, N** | 119 | 55 | 64 | - |
| **10** | **Diarrhoeal** | 9 (7.8) | 4 (7.6) | 5 (7.9) |  |
|  | **Normal** | 80 (69.0) | 31 (58.5) | 49 (77.8) |  |
|  | **Constipated** | 27 (23.2) | 18 (34.0) | 9 (14.3) | **0.04** |
|  | **Total, N** | 116 | 53 | 63 | - |
|  | ***missing^b^*** | 3 (2.5) | 2 (3.6) | 1 (1.6) | 0.60 |
| **20** | **Diarrhoeal** | 7 (6.3) | 3 (5.9) | 4 (6.6) |  |
|  | **Normal** | 95 (84.8) | 40 (78.4) | 55 (90.2) |  |
|  | **Constipated** | 10 (8.9) | 8 (15.7) | 2 (3.3) | 0.07 |
|  | **Total, N** | 112 | 51 | 61 | - |
|  | ***missing^b^*** | 7 (5.9) | 4 (7.3) | 3 (4.7) | 0.70 |
| **40** | **Diarrhoeal** | 5 (4.6) | 3 (6.3) | 2 (3.3) |  |
|  | **Normal** | 95 (88.0) | 38 (79.2) | 57 (95.0) |  |
|  | **Constipated** | 8 (7.4) | 7 (14.6) | 1 (1.7) | **0.02** |
|  | ***missing^b^*** | 11 (9.2) | 7 (12.7) | 4 (6.3) | 0.34 |
|  | **Total, N** | 108 | 48 | 60 | - |

***^a^*** Percent on non-missing values; ***^b^*** Percent on total number of patients; ***^c^*** Fisher’s exact test;

| **Table S4.** Adverse Events type frequency distribution by treatment arm and time | | | | |
| --- | --- | --- | --- | --- |
|  |  |  | **Treatment, N (%) *^a^*** | |
| **Days from baseline** | **Adverse Event** | **All patients**  **N (%)** | **HA-SSD** | **PeG** |
| **10** | Burning | 5 (4.3) | 3 (5.7) | 2 (3.1) |
|  | Erythema with blisters | 1 (0.9) | 0 | 1 (1.6) |
|  | Fecaloma | 1 (0.9) | 1 (1.9) | 0 |
|  | Rectal bleeding | 2 (1.7) | 2 (1.9) | 0 |
|  | Itching | 1 (0.9) | 1 (1.9) | 0 |
|  | Other*^b^* | 1 (0.9) | 0 | 1 (1.6) |
| **20** | Rectal bleeding | 2 (1.8) | 2 (3.9) | 0 |
|  | Itching | 3 (2.7) | 3 (5.9) | 0 |
|  | *Unspecified* | 1 (0.9) | 1 (1.9) | 0 |
| **40** | Itching | 2 (1.9) | 2 (4.3) | 0 |
|  | *Unspecified* | 1 (0.9) | 0 | 1 (1.6) |

***^a^*** Adverse Events percent on non missing values; *^b^* itching + burning + pain

**Table S5**. Summary of the repeated measures mixed models conducted on the average number of pills/patient (Paracetamol and Ketorolac). The models incorporated time, treatment, and the treatment-by-time interaction as fixed effects, with patient ID included as a random effect.

| **Mixed models** | *Mean pills/patient at 10 days from baseline* | *Treatment x Time* | *Treatment* | *Days from baseline* |
| --- | --- | --- | --- | --- |
| *Average number of pills/patient* | | | | |
| Paracetamol | p < 0.001 | p = 0.30 | p = 0.76 | p < 0.001 |
| Ketorolac | p < 0.001 | p = 0.02 | p = 0.28 | p < 0.001 |
